# Using Conventional Cameras as Sensors for Estimating Confidence Intervals for the Speed of Vessels from Single Images

**DOI:** 10.3390/s22114213

**Published:** 2022-06-01

**Authors:** Jose L. Huillca, Leandro A. F. Fernandes

**Affiliations:** Instituto de Computação, Universidade Federal Fluminense (UFF), Avenida General Milton Tavares de Souza, s/n, Niterói 24210-346, RJ, Brazil; laffernandes@ic.uff.br

**Keywords:** vessel speed, Kelvin wake, ship wake, error propagation, object detection, planar homography, metrology

## Abstract

In this paper, we describe an image-based approach for estimating the speed of a moving vessel using the wakes that remain on the surface of water after the vessel has passed. The proposed method calculates the speed of the vessel using only one RGB image. In this study, we used the vanishing line of the mean water plane, the camera height concerning the level of the tide, and the intrinsic parameters of the camera to perform geometric rectification on the surface plane of the water. We detected the location of troughs on one of the wake arms and computed the distance between them in the rectified image to estimate the speed of the vessel as a so-called inverse ship wake problem. We used a radar that was designed to monitor ships to validate the proposed method. We used statistical studies to determine the reliability and error propagation of the estimated values throughout the calculation process. The experiments showed that the proposed method produced precise and accurate results that agreed with the actual radar data when using a simple capture device, such as a conventional camera.

## 1. Introduction

Radar-based electronic navigation systems (ENSs) that keep the pilot informed about the location and speed of nearby vessels have been a significant technological advance for the maritime industry. Unfortunately, radars and other sensors may fail to detect stealth ships and non-metallic targets because they reflect a low amount of radiation. Additionally, many vessels are not equipped with an ENS. As a result, naked eye visibility still plays a key role in making decisions, especially at close range. Going further, projects have been carried out on the creation of autonomous vessels [1,2], in which autonomous navigation makes use of motion flow techniques, such as FeatFlow [3], as auxiliary sources of information.

Vessel detection and tracking using computer vision-based systems are convenient methods for measuring vessel speed [4]. A large number of algorithms estimate the vessel speed by analyzing the traces that are left by the vessel using synthetic-aperture radar (SAR) imagery [5,6]. Techniques, such as the CRSN [7], have been used to improve the quality of SAR images and CenterNet++ [8] performs vessel detection. In the work developed by Liu et al. [9], ship wakes were used to detect vessels and to identify the position and direction of the vessel using optical images. Although these techniques provide good results, the SAR and optical images that were considered in the above-mentioned studies were not captured at close range; they were generated by airborne sensors or satellites and could not be obtained from inside an autonomous vessel.

Other techniques perform tracking and estimations of speed using image sequences that were taken with a digital camera [10,11,12]. However, video-based techniques require fixed cameras to estimate speed from the relative motion. Therefore, this type of method is limited to applications in which the camera is onshore, since cameras that are placed on autonomous vessels and drones produce additional movement in the optical flow.

In a recent study, Huillca and Fernandes [13] presented a semi-automatic method to calculate the speed of a vessel directly from one image that was acquired by a conventional camera. The method is based on projective geometry and estimates vessel speed from an analysis of the Kelvin wake pattern. The key observation is that naval objects leave traces of their movements on the surface of the water and the appearance of these traces is related to speed. Actually, as Lord Kelvin demonstrated in 1887, the wakes that are left by vessels maintaining a constant course and speed can be modeled as a function of speed [14]. The inverse ship wake problem consists of estimating parameters, such as speed, from the observation of wake patterns [15]. The approach that was proposed by Huillca and Fernandes [13] detects features (crests or troughs) of the extracted wave arm and applies the inverse ship wake problem to estimate the speed of the corresponding vessel. However, the technique requires the horizon line to be visible in the image. The curves that are adjusted to the wave arms are also sensitive to noise and the analysis of the results of the study was limited to a few test cases using ground truth that was based on rough estimates of the maximum and minimum speeds that were reached by the observed vessels.

This paper presents an extended version of the approach that was proposed by Huillca and Fernandes [13]. The extension included the automatic estimation of the vanishing line, a different approach to identifying the wave arms, the use of the troughs in the closest wave arm to estimate the wavelength, the use of radar to validate the results, the estimation of confidence intervals for the measurements, and the analysis of error propagation throughout the computational chain. Unlike previous work, the use of a more sophisticated vanishing line detection method allowed for the application of the new method even when the line between the sky and the water was not visible. Our new clustering strategy for identifying the two wave arms made our approach less prone to misidentification. In the previous study, the validation was performed using only the speed information that was presented on the Rio de Janeiro Ferry Services website. In this work, we verified the accuracy and reliability of the measurements that were obtained through our method using ground truth data that were collected from a radar that was designed to monitor vessels, including ferries and other types of vessels. We also used a sampling-based method and first-order error propagation [16] to estimate the uncertainty of the estimations that were produced using our technique. We showed that the confidence intervals that were obtained with error propagation were equivalent to those that were computed by sampling, but also had the advantage of allowing for uncertainty estimation when using one image. Our results were consistent and agreed with the values that were obtained by the radar and did not present the limitations of video-based approaches, since all estimates were performed using a single image.

## 2. Computing Vessel Speed

**Figure 1:**
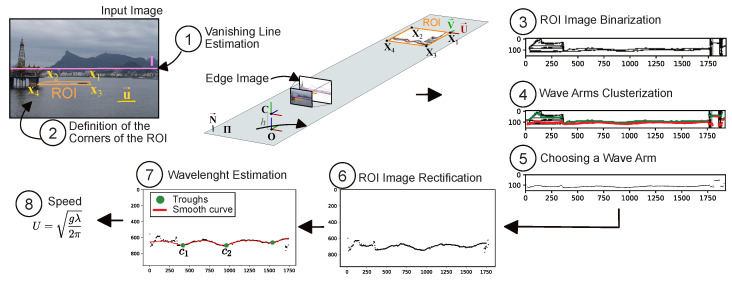
The pipeline followed to estimate the vessel speed. We intentionally flipped the rectified region of interest (ROI) image to make the vessel travel to the left.

For the proposed method, it is assumed that the camera is mounted at a given height *h* above sea level in such a way that the target ship and the traces that are left by the target ship can be observed (see Figure 1). The camera could be onshore, aboard a vessel or on a moving drone. The orientation of the camera in world space is estimated during the process. The speed is computed using the wavelength of the trace information. For each acquired image, the processing steps include: (i) the estimation of the vanishing line of the mean water plane; (ii) the definition of the corners of the region of interest (ROI), including the ship wake; (iii) the identification of the wave arms and troughs that are present in the ROI; and (iv) the estimation of the speed of the vessel using the wavelength.

The notation adopted in this article was inspired by Hartley and Zisserman [17] and is shown in Table 1.

### 2.1. Vanishing Line Estimation

We used the Horizon Line in the Wild (HLW) algorithm [18] to automatically detect the vanishing line of the water body. The HLW algorithm estimated the left- and right-hand endpoints of the vanishing line of the most prominent plane that was observed in the input color image I. In our equations, we represented the endpoints using homogeneous coordinates as vectors $p_{l}={\left(0,y_{p_{l}},1\right)}^{T}$ and $p_{r}={\left(W-1,y_{p_{r}},1\right)}^{T}$, where *W* is the width of I and $y_{p_{l}}$ and $y_{p_{r}}$ are the vertical coordinates of the points that were returned by the HLW algorithm. The vector $l={\left(A,B,C\right)}^{T}$ that encoded the vanishing line of the mean water plane in homogeneous coordinates could be computed as the cross product of vectors $p_{l}$ and $p_{r}$ [17]:

(1) $$l={\left(A,B,C\right)}^{T}=p_{l}\times p_{r}={\left(y_{p_{l}}-y_{p_{r}},W-1,\left(1-W\right)y_{p_{l}}\right)}^{T},$$

where *A*, *B*, and *C* are the coefficients of the general equation of the line Ax+By+Cw=0.

It is important to emphasize that the horizon line did not need to be visible for the HLW algorithm to estimate the endpoints of the vanishing line of the mean water plane. For instance, notice in Figure 1 that the horizon line would be behind the mountains. For the RANSAC-based technique that was used by Huillca and Fernandes [13], on the other hand, the horizon line between the sky and the water must be visible because it is detected as the most apparent straight line in the edge image.

### 2.2. Definition of the Corners of the ROI

The ROI had to include the ship wake. It was defined in I as the quadrilateral that resulted from the projection of a rectangular region onto the water surface (see Figure 1). We found the set of corners ${\left\{x_{k}\right\}}_{k=1}^{4}$ of the ROI in I using the reference corner $x_{1}={\left(x_{x_{1}},y_{x_{1}},1\right)}^{T}$, the direction $\vec{u}={\left(x_{\vec{u}},y_{\vec{u}},0\right)}^{T}={\left(\cos\phi_{\vec{u}},\sin\phi_{\vec{u}},0\right)}^{T}$ of the vessel in the image, the vanishing line l of the water (1), the camera calibration matrix K, the ROI size $W_{ROI}\times H_{ROI}$ (in meters) in 3D space, and the camera height *h* above sea level.

Using techniques such as YOLO [19] and the training model that was used by Breitinger et al. [20] to detect the vessels is straightforward. However, as this was a separate problem that was subject to the quality of the detection technique without the loss of generality, we chose to manually set $x_{1}$ and $\vec{u}$ in image space in our experiments. The selection condition for $x_{1}$ was to place this point in image space to the left of the vessel in 3D space. It could be placed either near the bow or near the stern. We set $x_{1}$ close to the bow for all images in our experiments. The definition of direction $\vec{u}$ in image space was straightforward. It could be defined by tracing a line segment from the stern to the bow or it could be calculated from the edge pixels of the wake as the eigenvector with the largest eigenvalue pointing to the bow. In this work, we extracted metadata from the image files to compute K. $W_{ROI}$ and $H_{ROI}$ were constant values that were defined by the user.

The camera height *h* was given by the construction and was defined as the distance between the camera and the mean water plane in meters. We assumed that the origin O of the world coordinate system (see Figure 1) lay on the orthogonal projection of the camera center C to the mean water plane Π, that the *X* and *Y* axes spanned Π, and that the *Z* axis was parallel to the vector $\vec{N}$ that was normal to Π. We set $C={\left(0,0,h,1\right)}^{T}$ and computed its orientation with respect to the world’s frame from the vanishing line l. Therefore, even though the camera could move with six degrees of freedom, only one (the height) had to be known a priori.

When a camera is mounted on a vessel’s mast, its height *h* can be calculated from the mast’s height and its relative orientation to the normal vector $\vec{N}$ (computed from the vanishing line). For drones, the vehicle needs to be able to determine its altitude. In our experiments, the camera was mounted on a tripod that was inside a building. In this case, the camera height above the sea level was calculated as the sum of the heights $h_{g}$, $h_{f}$, $h_{t}$, and $h_{s}$ representing the ground height, floor height, tripod height, and tide height, respectively:

(2) $$h=h_{g}+\delta h_{f}+h_{t}-h_{s},$$

where δ is the floor number on which the camera was mounted.

We let ${\left\{X_{k}\right\}}_{k=1}^{4}$ be the set of corners of the ROI lying on plane Π. By tracing a ray from the camera to Π through point $x_{1}$, we computed:

(3) $$X_{1}={\left(X_{X_{1}},Y_{X_{1}},0,1\right)}^{T}={\left(-hX_{\vec{D}}/Z_{\vec{D}},-hY_{\vec{D}}/Z_{\vec{D}},0,1\right)}^{T},$$

where $\vec{D}={\left(X_{\vec{D}},Y_{\vec{D}},Z_{\vec{D}}\right)}^{T}=M^{-1}x_{1}$ is the direction of the traced ray, M=KR, and R is a rotation matrix whose columns correspond to the *X*, *Y*, and *Z* axes of the camera in the world’s frame of reference (the red, green, and blue segments leaving C in Figure 1, respectively). The columns of R were ${\vec{R}}_{1}={\vec{R}}_{3}\times {\vec{R}}_{2}$, ${\vec{R}}_{2}=\mathrm{unit}\left({\left(1,0,0\right)}^{T}\times {\vec{R}}_{3}\right)$, and ${\vec{R}}_{3}=\mathrm{unit}\left(\mathrm{up}\left(K^{T}l\right)\right)$. The unit function normalized the vector to unit length and the up function changed the orientation of ${\vec{R}}_{3}$ when its $Y_{{\vec{R}}_{3}}$ coordinate was negative. It was necessary to correct the hand of the camera coordinate system by forcing the ${\vec{R}}_{2}$ vector to point upward, as with the normal vector $\vec{N}$ of the mean water plane and the *Z* axis of the world’s frame of reference (see Figure 1), while ${\vec{R}}_{1}$ and ${\vec{R}}_{3}$ pointed to the right and the front, respectively.

$X_{2}$, $X_{3}$, and $X_{4}$ were computed by translating $X_{1}$ by $W_{ROI}$ and $H_{ROI}$ in directions $\vec{U}=\mathrm{unit}\left(A\vec{E}\right)$ and $\vec{V}=B\vec{U}$ on plane Π:

(4a) $$\begin{array}{cc} X_{2} & =X_{1}-W_{ROI}\vec{U}, \end{array}$$

(4b) $$\begin{array}{cc} X_{3} & =X_{1}-H_{ROI}\vec{V}, \end{array}$$

(4c) $$\begin{array}{cc} \;\;\;\;\;\;\;\;X_{4} & =X_{1}-W_{ROI}\vec{U}-H_{ROI}\vec{V}, \end{array}$$

where $\vec{U}$ is the direction of the vessel in world space and $\vec{V}$ is the perpendicular direction that was computed by rotating $\vec{U}$ in Π through an angle of π/2. Thus, B was a constant rotation matrix. Here, $\vec{E}={\left(X_{\vec{E}},Y_{\vec{E}},Z_{\vec{E}}\right)}^{T}=M^{-1}\vec{u}$ was the back projection of $\vec{u}$ (an improper point) onto the world coordinate system and A encoded the orthogonal projection onto Π.

Finally, the corners of the ROI in I were computed as:

(5) $$x_{k}=PX_{k},$$

where k∈{2,3,4}, $P=M\left(\begin{array}{c} I|-\tilde{C} \end{array}\right)$ is the 3×4 camera matrix, I is a 3×3 identity matrix, and $\tilde{C}={\left(0,0,h\right)}^{T}$.

### 2.3. Finding the Wave Arms

We used the edge image B of the color image I to find the wave arms. To avoid processing the whole image or working with a non-rectangular ROI, the procedure for finding the wave arms considered a small portion of B that was defined as the axis-aligned bounding box of the ROI. Thus, in the remainder of this section, all described processing was restricted to that portion of B.

In this work, we used the Richer Convolutional Features (RCF) algorithm [21] to compute the edge image B from I. RCF helped to detect the wakes that were left by the vessels by making the waves more visible. Among the edge detection strategies that we tested, RCF was less sensitive to weather conditions and poor natural light.

**Figure 2:**
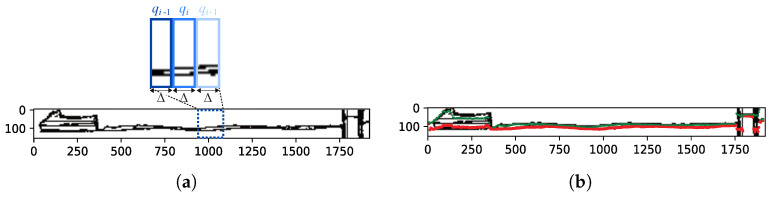
Finding the wave arms: (**a**) we applied the *k*-means algorithm to the edge pixels that were included in each partition $q_{i}$ of the ROI’s bounding box and the partitions each had a width of Δ pixels; (**b**) the detected wave arms.

The edge image B that was produced by RCF was an intensity image, in which 1 indicated that the pixel had a high chance of being an edge while 0 meant the opposite. We used the Otsu algorithm to find an automatic threshold *t* to separate the pixels into the two classes, i.e., edge and non-edge, and compute the binary image $B^{*}$. We used the *k*-means algorithm [22] with k=2 to differentiate the two wave arms that were present in the wakes that were left by the ships. The image coordinates of the edge pixels were taken as the inputs for the algorithm. As illustrated in Figure 2a, the *k*-means algorithm was not directly applied to the entire binary image. We divided the portion of $B^{*}$ that was inside the bounding box of the ROI into vertical partitions $q_{i}$, each with a width of Δ pixels and height that was equal to the height of the bounding box. In each partition $q_{i}$, we applied the *k*-means algorithm to obtain the centroid of each wake arm. The *k*-means points in partition $q_{i}$ were equal to the centroids that were calculated in $q_{i-1}$, except for the first partition (i=1) or when no centroid was detected in $q_{i-1}$. For those cases, the two points were taken as random. The points that were obtained as the centroids defined the samples for the curves that described each wake arm. Figure 2a highlights three consecutive partitions, while Figure 2b shows the detected centroids using green and red for the most distant and closest wake arms, respectively. In our experiments, we set Δ to 5 pixels. According to our experience, the results of the curve fitting process that follows did not differ when a Δ value between 3 and 9 pixels was chosen.

### 2.4. Wavelength and Speed Estimation

To solve the inverse Kelvin wake problem, we needed to calculate the Euclidean distance between at least two consecutive troughs or crests in the world coordinate system. A key observation is that the image of the wave arm that was closest to the camera was the least affected by the turbulence of the wake. In addition, its troughs were less affected by errors when we performed the rectification of the ROI. The steps for performing wavelength and speed estimation are described below.

#### 2.4.1. ROI Image Rectification

**Figure 3:**
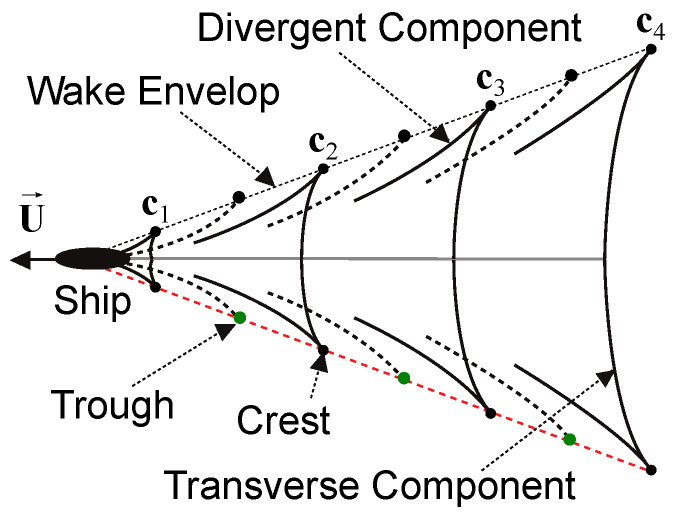
The Kelvin wake structure, indicating the transverse and divergent components as well as the crests and troughs of the wave arms.

The objective of the ROI rectification was to eliminate the projective distortion that was introduced by the camera from the image of the mean water plane, thereby simulating an aerial view of the ROI that was similar to the sketch that is presented in Figure 3. We used the line at infinity $l_{\infty }$ of the water plane to remove affine distortion and the camera height above sea level to eliminate projective ambiguity. The line at infinity $l_{\infty }$ allowed for the recovery of the related properties of image elements, such as parallelism and the proportion of areas [17].

For convenience, we used canonical coefficients to define the general equation of the line at infinity, i.e., $l_{\infty }={\left(0,0,1\right)}^{T}$ in homogeneous coordinates. By taking the vanishing line $l={\left(A,B,C\right)}^{T}$ from (1), the projective transformation H that mapped l onto $l_{\infty }$ was given by:

(6) $$H=H_{A}H_{l}=\left(\begin{array}{ccc} a_{11} & a_{12} & a_{13}\\ a_{21} & a_{22} & a_{23}\\ 0 & 0 & 1 \end{array}\right)\left(\begin{array}{ccc} 1 & 0 & 0\\ 0 & 1 & 0\\ A & B & C \end{array}\right).$$

Using H, we were able to rectify each point on the mean water plane in the image. In (6), $H_{l}$ was used to offset the affine matrix $H_{A}$, in which $a_{11}$, $a_{12}$, $a_{21}$, and $a_{22}$ defined a 2×2 non-singular matrix and ${\left(a_{13},a_{23}\right)}^{T}$ was a translation vector.

A planar affine transformation $H_{A}$ has six degrees of freedom, which can be computed using three-point correspondences. We used the correspondence between the corners of the rectified ROI using homogeneous coordinates ${\left(0,0,1\right)}^{T}$, ${\left(W_{ROI},0,1\right)}^{T}$, and ${\left(0,H_{ROI},1\right)}^{T}$ and points $x_{k}^{\prime }=H_{l}x_{k}$ for k∈{1,2,3}, where $x_{k}$ are the corners of the ROI in image space (5). From the correspondences, we defined a linear system of the equation in matrix form:

$$A\;\vec{v}=\left(\begin{array}{ccccccc} x_{x_{1}^{\prime }} & y_{x_{1}^{\prime }} & w_{x_{1}^{\prime }} & 0 & 0 & 0 & 0\\ 0 & 0 & 0 & x_{x_{1}^{\prime }} & y_{x_{1}^{\prime }} & w_{x_{1}^{\prime }} & 0\\ x_{x_{2}^{\prime }} & y_{x_{2}^{\prime }} & w_{x_{2}^{\prime }} & 0 & 0 & 0 & -W_{ROI}\\ 0 & 0 & 0 & x_{x_{2}^{\prime }} & y_{x_{2}^{\prime }} & w_{x_{2}^{\prime }} & 0\\ x_{x_{3}^{\prime }} & y_{x_{3}^{\prime }} & w_{x_{3}^{\prime }} & 0 & 0 & 0 & 0\\ 0 & 0 & 0 & x_{x_{3}^{\prime }} & y_{x_{3}^{\prime }} & w_{x_{3}^{\prime }} & -H_{ROI} \end{array}\right)\left(\begin{array}{c} v_{11}\\ v_{12}\\ v_{13}\\ v_{21}\\ v_{22}\\ v_{23}\\ v_{33} \end{array}\right)=\left(\begin{array}{c} 0\\ 0\\ 0\\ 0\\ 0\\ 0\\ 0 \end{array}\right),$$

where A is a matrix of known values and $\vec{v}$ is the vector of variables. The coefficients of $H_{A}$ were computed as $a_{ij}=v_{ij}/v_{33}$ after solving the system for $\vec{v}$ as the right-hand null space of A. We used the singular value decomposition $A=USV^{T}$ to solve the system by taking $\vec{v}$ (i.e., the vector that was associated with the zero singular value) as the last column of matrix V.

**Figure 4:**
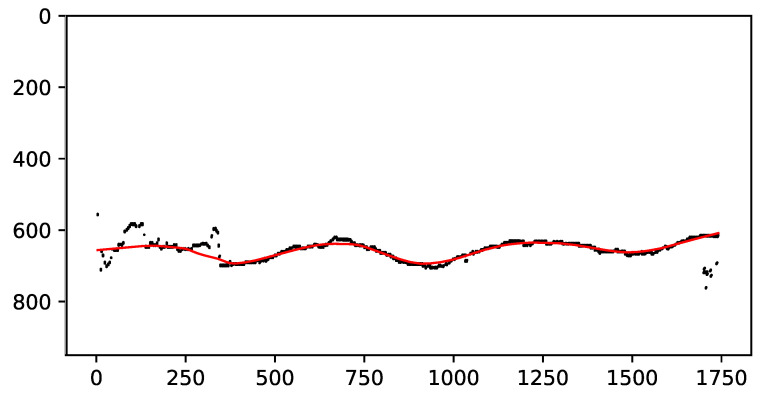
The black points are the curve samples and the red line is the smooth curve that was computed by the LOWESS algorithm [23]. Axes in centimeters.

By construction, the wave arm that was closest to the camera was always as shown below in the ROI image. Therefore, the rectification only needed to be applied to the points that were obtained in Section 2.3 for that wave arm, since we were interested in the red line that is presented in Figure 3. The black dots in Figure 4 represent the rectified set of points that was used to fit the red curve in Figure 2.

#### 2.4.2. Curve Fitting

As can be seen in the black points in Figure 4, the discrete set of points that was obtained in Section 2.3 could be corrupted by noise, which made it challenging to find the troughs of the wake. We used the locally weighted scatterplot smoothing (LOWESS) algorithm [23] to smooth the digital curve that was extracted from the rectified ROI image. The black points in Figure 4 correspond to the input curve samples in this example, while the red line is the resulting smooth curve that was used to find the troughs.

Huillca and Fernandes [13] took the point samples for one of the wave arms and followed a naive approach that used a Savitzky–Golay filter [24] for curve fitting. They performed both procedures in the rectified image of the ROI. According to our experience, the use of the *k*-means algorithm on the input image domain to obtain the point samples followed by the application of the LOWESS algorithm to the points that were mapped onto the domain of the rectified ROI was much less sensitive to noise.

#### 2.4.3. Wavelength Estimation

The wavelength of the transverse components of the Kelvin wake pattern (see Figure 3) can be estimated using the distance between the successive crests or troughs. From those wavelengths, the speed of a vessel can then be estimated [5]. One finds a set of crests/troughs as the curve maximum or minimum, depending on the direction of the vessel with respect to the camera. Since we had a curve that corresponded to the arm that was closest in the V-shaped pattern, we could find the crests and troughs as follows:

- When the vessel went to the right in the input image I, the curve maximum and minimum corresponded to the troughs and crests of the wave arm;
- When the vessel went to the left in the input image I, the curve maximum and minimum corresponded to the crests and troughs of the wave arm.

We avoided the identification of noisy troughs by imposing a minimum horizontal distance of ψ=20 m between valid consecutive troughs and by only extracting two minimums and maximums (depending on the case). The value of ψ was empirically defined based on the observation that the speed is close to 10 knots for a wavelength of approximately 20 m and, as discussed in Section 4, most vessels that leave visible tracks move at higher speeds. We computed the wavelength λ by replacing *D* in:

(7) $$\lambda =\frac{\sqrt{3}}{2}D,$$

where $D={\mathrm{dist}}_{E}\left(c_{n},c_{n+1}\right)$ is the Euclidean distance (in meters) between the location of the crests (or troughs) $c_{n}$ and $c_{n+1}$.

#### 2.4.4. Vessel Speed Estimation

Finally, the speed of the vessel was:

(8) $$U=1.944\sqrt{\frac{g}{2\pi }\lambda },$$

were λ is given by (7) and g≈ 9.80665 m/s^2^ is the acceleration of gravity. Since knot is the unit of measurement that is used for speed in maritime navigation, the m/s values had to be multiplied by 1.944 to be converted into knots.

## 3. First-Order Error Propagation

The theory of errors [16] provides the expressions that were needed to estimate the standard uncertainty of a measurement *U* using the standard uncertainties of the experimental values in the dataset $\vartheta =\{y_{p_{l}},y_{p_{r}},x_{x_{1}},y_{x_{1}},\phi_{\vec{u}},h_{g},h_{f},h_{t},h_{s}\}$. In matrix form, the first-order error propagation of such uncertainties was expressed by:

(9) $$\sigma_{U}^{2}=\nabla_{U}\Lambda_{\vartheta }\nabla_{U}^{T},$$

where

(10) $$\nabla_{U}=\left(\frac{\partial U}{\partial y_{p_{l}}},\frac{\partial U}{\partial y_{p_{r}}},\frac{\partial U}{\partial x_{x_{1}}},\frac{\partial U}{\partial y_{x_{1}}},\frac{\partial U}{\partial \phi_{\vec{u}}},\frac{\partial U}{\partial h_{g}},\frac{\partial U}{\partial h_{f}},\frac{\partial U}{\partial h_{t}},\frac{\partial U}{\partial h_{s}}\right)$$

is the Jacobian matrix of the function that calculated the speed *U* of a vessel using the method that was described in Section 2 and

(11) $$\Lambda_{\vartheta }=\mathrm{diag}\left(\sigma_{y_{p_{l}}}^{2},\sigma_{y_{p_{r}}}^{2},\sigma_{x_{x_{1}}}^{2},\sigma_{y_{x_{1}}}^{2},\sigma_{\phi_{\vec{u}}}^{2},\sigma_{h_{g}}^{2},\sigma_{h_{f}}^{2},\sigma_{h_{t}}^{2},\sigma_{h_{s}}^{2}\right)$$

is the covariance matrix that encoded the uncertainty of the input variables that were used to compute *U*. In this paper, we assumed the independence of the input variables. Thus, their covariances were zero, with $\Lambda_{\vartheta }$ being a diagonal matrix and $\sigma_{\theta }$ being the standard deviation of the input variable θ∈ϑ. The partial derivatives in $\nabla_{U}$ (10) were taken with respect to the nine variables in ϑ. Appendix A presents the expressions that were used to compute those partial derivatives.

**Figure 5:**
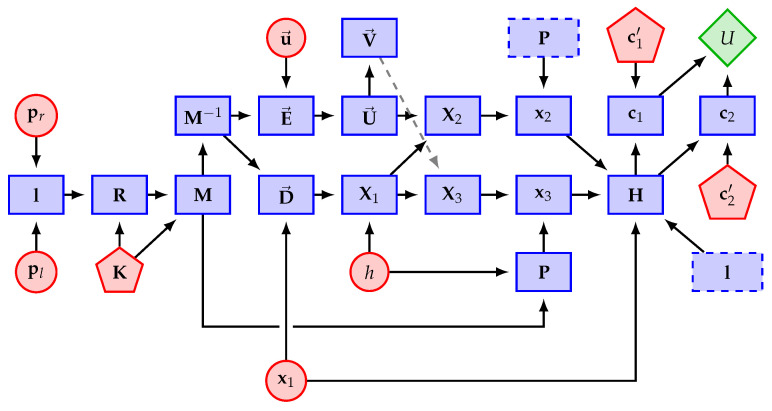
Computational chain for estimating vessel speed (rhombus) using experimental variables with (circles) and without (pentagons) uncertainty.

The computational flow of the *U* function is illustrated in Figure 5, in which the circles represent the input variables with uncertain values, pentagons represent the input variables that we assumed to have no uncertainty, rectangles represent the intermediary variables, and the rhombus is the estimated speed of the vessel. Altogether, the proposed method has 20 input variables:

$y_{p_{l}}$, $y_{p_{r}}$: The *y* axis coordinates of the endpoints $p_{l}$ and $p_{r}$ of the vanishing line that are estimated for the mean water plane, respectively (Section 2.1);
$x_{x_{1}}$, $y_{x_{1}}$: The coordinates of the corner $x_{1}$ of the ROI in the input image I;
$\phi_{\vec{u}}$: The angle that defines the direction $\vec{u}$ of the vessel in the input image I;
$h_{g}$, $h_{f}$, $h_{t}$, $h_{s}$: The set of heights that is used in (2) to calculate the camera height *h* above the sea level in meters;
$x_{c_{1}^{\prime }}$, $y_{c_{1}^{\prime }}$, $w_{c_{1}^{\prime }}$, $x_{c_{2}^{\prime }}$, $y_{c_{2}^{\prime }}$, $w_{c_{2}^{\prime }}$: The homogeneous coordinates of two adjacent troughs of the wave arm that is closest to the camera, i.e., points $c_{1}$ and $c_{2}$ that were used in (7), but represented by pixel coordinates in I. These variables are not taken as sources of uncertainty because their rectified counterparts ($c_{1}={\left(x_{c_{1}},y_{c_{1}},1\right)}^{T}$ and $c_{2}={\left(x_{c_{2}},y_{c_{2}},1\right)}^{T}$) naturally include the uncertainty that is propagated from other variables (see Appendix A for details);
$\alpha_{x}$, $\alpha_{y}$, γ, $x_{o}$, $y_{o}$: The intrinsic parameters that define the camera calibration matrix K. They are the focal length in terms of pixel dimensions in the *x* and *y* directions, skew, and the coordinates of the principal point in terms of pixel dimensions, respectively [17]. Recall from Section 2.2 that we extracted metadata from the input image file to compute K. In this work, we assumed that the intrinsic parameters of the camera were constant values since it was observed that they do not usually have much influence on the uncertainty of image-based measurements [25].

Section 4 describes how to estimate the uncertainty in θ∈ϑ.

## 4. Experiments and Results

The procedures that were described in Section 2 were implemented in Python 2.7.0. Speeds were calculated on a computer that had an Intel Xeon CPU E-2698 v4 with 2.20 GHz and a Tesla P100-SXM2 video card with 16 GB of VRAM.

In the experiments, the images of moving vessels were acquired under natural lighting and different weather conditions. The images were taken using a Nikon D3300 camera with 24.2 megapixels and were then encoded in JPG format file (any other image format can be used without affecting the results). The lens model that we used was an AF-S DX NIKKOR, with an 18∼55 mm focal length and vibration reduction (VR II) [26]. The resolution of the captured images was 6000×4000 pixels. The ROI size lying on plane Π was intentionally set to $W_{ROI}\times H_{ROI}=180\times 90$ m in order to cover the wave arms of vessels that were traveling at more than 10 knots, as the speed of the vessels was rarely less than this threshold within the field of view that was used for the experiments. The camera was mounted in two places. The camera height was approximately h=27.79 and h=23.88 m for images i1–i17 and i18–i23, respectively. The camera height varied according to the tide height at the time the image was taken and the floor of the building.

**Figure 6:**
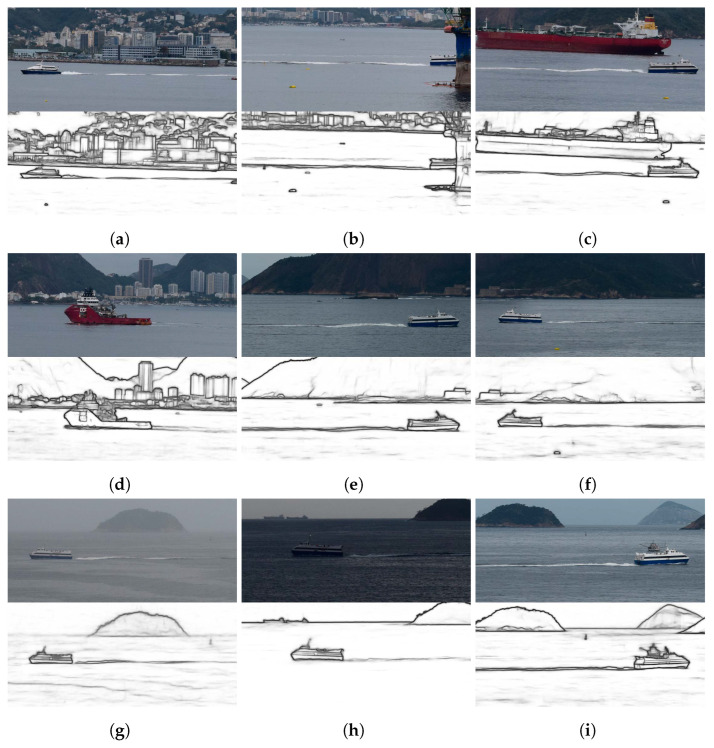
Cropped versions of some of the images that were used in the experiments: images i1, i9, and i10 show HSC passenger vessels; images i10, i13, i18, and i22 show MC25 passenger vessels; image i7 shows another vessel model. (**a**) Image i1; (**b**) Image i2; (**c**) Image i5; (**d**) Image i7; (**e**) Image i13; (**f**) Image i16; (**g**) Image i18; (**h**) Image i22; and (**i**) Image i17.

**Figure 7:**
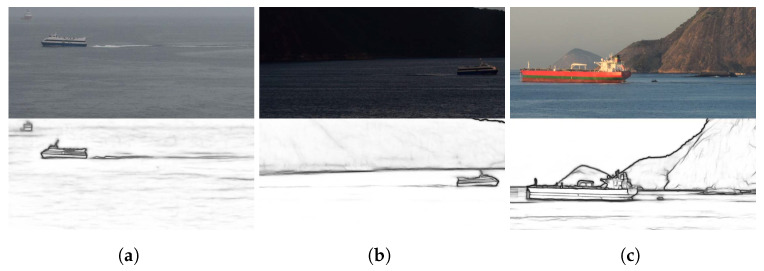
Cropped versions of some of the images in which our approach could not detect wave arms that had at least two well-defined troughs in the edge image.

A total of 40 images was obtained, of which 23 were used to analyze the results that are presented in this section. Table 2 describes the conditions under which the images that we used were acquired and Figure 6 shows some of the cropped versions. Special attention was paid to the noisy inputs that we included in our experiments, such as images i18 to i23 (see Figure 6g,h), because those cases included natural noise as they were acquired during a scattered storm or the partially cloudy and windy weather that followed the storm. The remaining 17 of the original 40 images were not considered because they were taken during unfavorable weather conditions (Figure 7a) and low natural lighting (Figure 7b), which prevented the edge detection approach from being successful in the detection of the troughs. Additionally, some of the vessels in those images were merchant ships (Figure 7c). As such, their speed had to be low because they were close to a port area. In all of those cases, at least two troughs of the traces that were left by the vessels could not be distinguished, even by human observers. In Table 2, the tide height was obtained from webpages that freely provide sea conditions [27,28,29]. The noise that was introduced by the wind speed was not considered in these experiments.

A radar that was designed to monitor vessels was used as a resource to validate the proposed method. The radar was a FAR−21×7 series of *X*- and *S*-bands with a 19-inch LCD screen [30]. The radar screen information was captured using a smartphone camera. The radar screen included the name and knot speed of the tracked vessel. The images that were taken of the radar screen and the moving vessel were acquired at approximately the same time. Table 2 shows the identification of the vessels (columns “Model” and “Name”), the time at which both pictures were taken (column “Time”), and the speed that was measured by the radar (column “*U*”). Of the 23 images that were used, 22 were of six different passenger vessels (models HSC and MC25) and one was a tugboat (image i7, Figure 6d). As we had limited access to the radar (upon authorization), it was necessary to restrict the image sections and only take 40 images. Additionally, later access was not allowed due to the COVID-19 pandemic. Even so, the set of images that was captured led to exciting results that prove the technique to be promising.

Considering that the data that were used as the input (e.g., the *y* coordinate of the endpoints of the vanishing line, the camera height, the reference corner of the ROI, and direction of the vessel in image space) were subject to errors, it was expected that the estimated speeds would also have uncertainties. By comparing the computed values to the speeds that were measured by the radar, it was possible to develop an idea of the accuracy and precision of the proposed technique. In Section 4.1, we analyze the relative error of our estimations and compare the quality of the estimates that were made using our technique to the approach that was presented by Huillca and Fernandes [13]. Section 4.2 and Section 4.3 present an analysis of the confidence intervals that were computed using sampling and first-order error propagation. In Section 4.4, Section 4.5 and Section 4.6, we discuss the influence of each experimental variable on the uncertainty of the estimated speed, the resilience to changes in the resolution of the input image, and the variations in JPG compression rate, respectively.

For Section 4.2, Section 4.3 and Section 4.4, the input uncertainties were estimated as follows:

$\sigma_{y_{p_{l}}}$, $\sigma_{y_{p_{r}}}$: The standard deviations of $y_{p_{l}}$ and $y_{p_{r}}$ were estimated using:
  (12) $$\sigma_{z}=\sqrt{\frac{1}{n-1}\sum_{i=1}^{n}{\left(z_{i}-\mu_{z}\right)}^{2}},$$
  where $\mu_{z}$ is the mean value of the differences $z_{i}$ between the *y* coordinates that were observed on endpoints that were returned by HLW and the endpoints of vanishing lines that were manually identified by us in a dataset comprising n=30 images, which led to $\sigma_{y_{p_{l}}}=22.72$ and $\sigma_{y_{p_{r}}}=23.62$ pixels;
$\sigma_{x_{x_{1}}}$, $\sigma_{y_{x_{1}}}$: We also used the n=30 experimental samples to set $\sigma_{x_{x_{1}}}=5.51$ and $\sigma_{y_{x_{1}}}=2.41$ pixels. The samples were obtained by repeatedly selecting the first corner of the ROI in the chosen image to serve as a reference. The standard deviations for the $x_{x_{1}}$ and $y_{x_{1}}$ coordinates were calculated using (12) for the coordinates of the selected points;
$\sigma_{\phi_{\vec{u}}}$: The same reference image was used to indicate the orientation of a vessel, which produced n=30 angular samples that were used to compute $\sigma_{\phi_{\vec{u}}}=0.011$ radians;
$\sigma_{h_{g}}$, $\sigma_{h_{f}}$, $\sigma_{h_{t}}$, $\sigma_{h_{s}}$: The standard deviations of the tripod height and the floor height were empirically set to $\sigma_{h_{t}}=0.006$ and $\sigma_{h_{f}}=0.03$ m, respectively, by assuming a conservative uncertainty for the measurement of those input variables. We used Google Maps to measure the ground height and set its uncertainty to $\sigma_{h_{g}}=1.15$ m based on the variations we observed in this tool. We estimated $\sigma_{h_{s}}=0.05$ m by applying (12) to a set of n=27 average tidal heights $z_{i}$ that were computed from observations in [27,28,29].

We assumed that the locations $c_{1}$ and $c_{2}$ of the troughs that were detected in the rectified ROI images carried the uncertainties that were introduced by the input variables, but the detection process itself did not introduce any new uncertainties (see Figure 5 and Appendix A).

### 4.1. Analysis of Relative Error

Relative error $\varepsilon_{r}=\varepsilon_{a}/U$ indicates the proportion of the absolute error $\varepsilon_{a}=\left|\hat{U}-U\right|$ of an estimated value $\hat{U}$ with regard to the true value *U*. We used $\varepsilon_{r}$ to determine the accuracy of our approach. In Table 2, the absolute error is given in knots. $\hat{U}$ was calculated by applying the proposed method, while *U* was measured by the radar.

We used the troughs of the closest wave arms to estimate the $\hat{U}$ values that are presented in Table 2. Those troughs were the least affected by the noise that was introduced by the vessel’s turbulence and the distortion of the rectified elements that were not in the actual mean water plane. According to Table 2, the relative error was below 2% for ten images, within the [2%,4%] interval in six cases, within the (4%,5%] range in three images, and between 5% and 10% in three cases. Only image i7 (Figure 6d) had a relative error that surpassed the true measure (109%). The explanation for this behavior is that the trace that was left by the tugboat was weak because it was traveling at 9.2knots and we set the ψ parameter for the minimum horizontal distance between valid consecutive troughs as 20 m, which limited the estimated speeds to a minimum of 10knots. The mean and median relative errors that are presented in Table 2 were 8.18% and 2.60%, respectively. The mean relative error was clearly affected by the result in image i7. Observing the robust statistics that were provided by the median, we could conclude that the proposed method was accurate.

To the best of our knowledge, the only work that has presented an approach for the estimation of the speed of moving vessels using single color images was developed by Huillca and Fernandes [13]. The subtable in Table 2 summarizes the results that were obtained by their approach after we replaced their RANSAC-based vanishing line detection scheme with the HLW algorithm. Otherwise, their method would not have been applicable to most of the images in Table 2. Unfortunately, it was not possible to perform a comparison between our approach and techniques that use image sequences, such as [10,11], because their implementation was not available and the descriptions that are presented in the articles proved to be insufficient for proper reproduction. In any case, such techniques cannot be used for video from cameras that are onboard vessels, which limits the scope of their application.

Observing $\varepsilon_{r}$ in Table 2, our new technique outperformed the previous approach for 20 out of the 23 images. For two cases in which our relative errors were higher, the difference in the errors was only 2.4% on image i15 and 3.3% on image i22. The third case was image i7, which, as previously mentioned, was not correctly handled by our approach because of the ψ parameter that was set.

### 4.2. Analysis of Confidence Intervals Estimated Using the Samples

In practice, each image that is presented in Table 2 provided one sample for which we could estimate speed. One way to assess the accuracy of the technique was to analyze the variations in the speed estimates that were obtained for the same vessel when considering a set of images that were captured under similar conditions as the input. To simulate several image captures of the same ship, we introduced small variations in the input variables that were considered to be sources of uncertainty for each captured image. We used the standard deviations that were assumed in the error propagation model (11) to produce n=150 Gaussian-distributed variations in the original set of input values for each image in Table 2, thereby generating samples from which we could compute speeds and the corresponding confidence intervals:

(13) $$CI\left(\gamma \right)=\left[\bar{U}-t_{\gamma }\frac{s_{U}}{\sqrt{n}},\bar{U}+t_{\gamma }\frac{s_{U}}{\sqrt{n}}\right],$$

where $\bar{U}$ is the mean speed of the sample, $s_{U}$ is the standard deviation of the sample, $t_{\gamma }$ is a *t*-Student variable with n−1 degrees of freedom, and γ is the confidence level.

**Figure 8:**
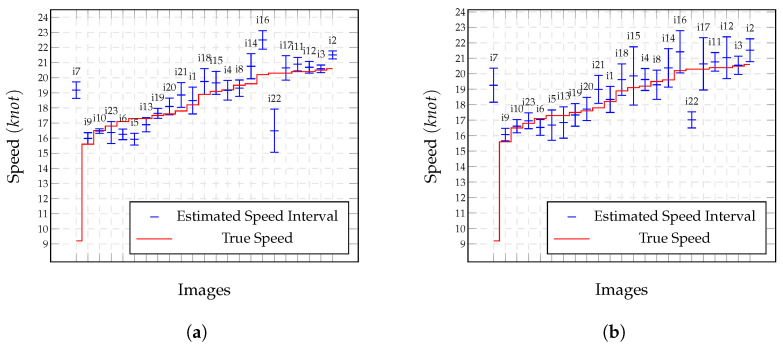
Confidence intervals (γ=99.8%) that were computed using (**a**) sampling and (**b**) first-order error propagation. Images sorted by vessel speed.

Figure 8a shows the confidence intervals with γ=99.8% that were calculated for the vessels using sampling (Table 2). The narrowest confidence intervals were for images i10 and i3, which were 0.30 and 0.50knots wide, respectively. Notice that the mean speed $\bar{U}$ was close to the true speed *U* in most cases and was included within the confidence interval in 16 out of the 23 images. Images i7, i16, and i22 were the cases with the most considerable distances between *U* and $\bar{U}$, whose confidence intervals did not include the true speed. The problem with image i7 was discussed in Section 4.1. For image i16 (Figure 6f), the location of the troughs was affected by the weather conditions. Notice the presence of more capillary wakes that were due to wind in this image than in the other images in Figure 6. For image i22 (Figure 6h), low natural lighting made the trail of the vessel very blurred. For the remaining four cases in which the true speed was outside of the confidence interval (images i6, i11, i14, and i21), the distance to the limits of the interval was negligible and ranged from 0.05 to 0.50knots.

Including all cases that are presented in Figure 8a, the largest confidence intervals were 2.86 and 1.78knots for images i1 and i22, respectively. The median interval was only 0.3knots. The variations in the values that were reported by the radar for the three consecutive speed measurements of vessels Zeus (images i19 to i21), Neptune (images i4 to i6), and *Missing* (images i11 to i13) were 0.3, 2.1, and 3.1knots, respectively. Thus, we could conclude that the proposed approach was accurate. However, we cannot make a strong statement in this regard because each interval was calculated using samples that were generated from one image.

### 4.3. Analysis of Confidence Intervals Estimated Using Error Propagation

In this section, we analyze the confidence intervals that were produced by the first-order error propagation approach, as discussed in Section 3, and compare them to the intervals that were produced using sampling.

First-order error propagation may provide the correct Gaussian uncertainty for the resulting estimations when the uncertainty of the input variables follows Gaussian distribution and the process for computing the resulting values is linear. Otherwise, it provides a first-order approximation of the error [16]. To verify which is the case for our approach, we used the Shapiro and Wilk [31] test to check whether the resulting samples that were produced in Section 4.2 fit Gaussian distribution. The null hypothesis of this test was: the data are normally distributed when ρ>α. For α=0.05, images i7, i16, i19, i22, and i23 had ρ-values that suggested evidence of non-normality. Therefore, we could only expect an approximation from the first-order error propagation of these cases.

The ratio $r=s_{U}/\sigma_{\hat{U}}$ between the standard deviations that were computed using sampling and propagation showed that the first-order error propagation approach was equivalent to and slightly more conservative than the sampling-based approach. The only exceptions were r>1 for images i1, i22, and i23. In 16 cases, 0.4≤r≤1. This was reflected in the results that are presented in Figure 8b, in which only two confidence intervals clearly did not include the true speeds (images i7 and i22) and four almost included them (images i2, i6, i9, and i21). Notice that images i7, i22, and i23 did not pass the normality test.

The narrowest intervals in Figure 8b were 0.80 and 0.86knots in width, while the widest were 3.38knots (image i17) and 3.76 knots (image i15). Using first-order error propagation, the mean width of the confidence intervals was 0.942knots and the median was 0.46knots.

With error propagation, it was easy to detect cases that had more considerable uncertainty for the calculated speeds because this approach does not require several samples.

### 4.4. Impact of the Uncertainty of Each Input Variable

The impact of each input variable on the uncertainty of the estimated speed could be assessed using the error propagation model (9). The absolute contribution of any input variable θ∈ϑ was obtained using a covariance matrix $\Lambda_{\vartheta }$ (11), for which the only non-zero elements were those related to θ in the main diagonal. The relative impact of θ was obtained by dividing its absolute impact by the sum of the absolute impacts of all input variables.

**Figure 9:**
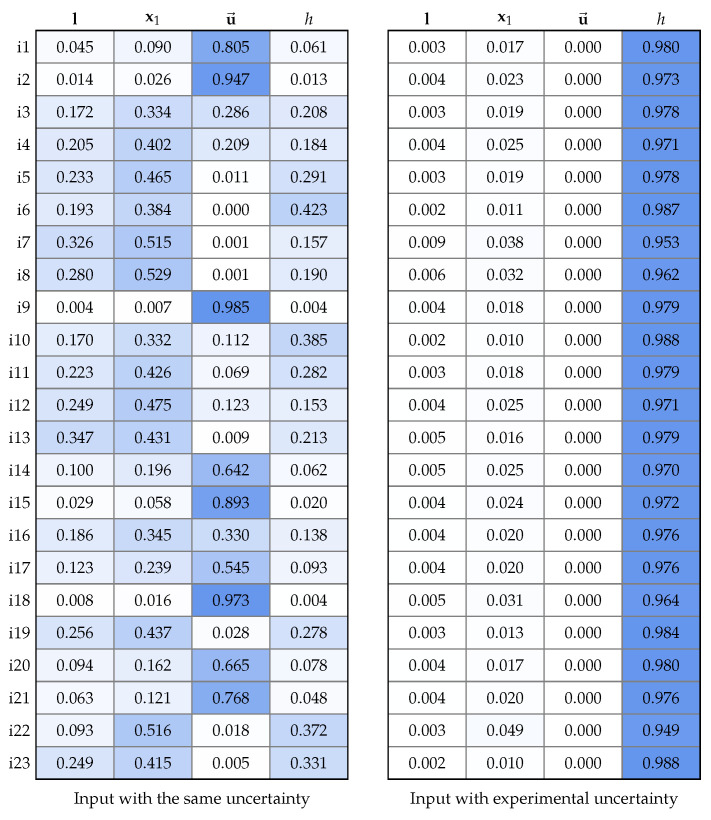
The relative impact of the input parameters on the computed speeds, assuming that (**left**) all input variables were independent and had the same uncertainty or (**right**) had the uncertainty that was estimated for the experiments: l represents the vanishing line; $x_{1}$ is the reference corner of the ROI; $\vec{u}$ is the direction of the vessel; and *h* is the camera height. Stronger shades of blue indicate greater relative impact.

For this analysis, we grouped the input variables into four groups (see the columns of the heatmap tables in Figure 9): the first group l included $y_{p_{l}}$ and $y_{p_{r}}$; the second group represented $x_{1}$ and included $x_{x_{1}}$ and $y_{x_{1}}$; the third group was $\vec{u}$ and only included the angle $\phi_{\vec{u}}$; and the last group included the variables $h_{g}$, $h_{f}$, $h_{t}$, and $h_{s}$, which were used to compute the camera height *h*.

Figure 9 (left) shows the relative impact of each group of input variables, assuming that they all had the same uncertainty ($\sigma_{\theta }=1$ for all θ∈ϑ) and were independent. Taking this as a premise, the largest sources of uncertainty for the estimated speeds were the parameters of $x_{1}$ and $\vec{u}$. One possible explanation is that $x_{1}$ and $\vec{u}$ were the variables that were used to calculate the first point $X_{1}$ in the world coordinate system and $X_{1}$ was used to calculate the remaining corners. Furthermore, the ROI coordinates in image space came from the world coordinate system and they directly fed the uncertainties into the matrix H.

Figure 9 (right) shows the scenario illustrating the results that were obtained by the estimated uncertainties of the input variables for the proposed method. It can be seen that the most significant source of uncertainty was the camera height *h*, which demonstrates the importance of this parameter in breaking projective ambiguity and, consequently, the correct scale of the estimates that are produced. In the particular case of our experiments, it was necessary to assume $\sigma_{h_{g}}=1.15$ since we observed inconsistent height readings at close points on the map.

### 4.5. Resilience to Variations in Resolution

In this experiment, the tests were carried out with scaled versions of the original image. This experiment simulated the use of lower resolution cameras and the speed estimation of more distant vessels. Here, we discuss the results that assumed scaling factors of 0.5, 0.25, and 0.125. In each case, the camera calibration matrix K and the location of the reference corner $x_{1}$ of the ROI were transformed accordingly. Furthermore, the corresponding edge image and the new *y* coordinates for the endpoints of the vanishing line l were obtained by applying the RCF and HLW algorithms to the scaled versions of the input images. The camera height *h* and the direction of the vessel $\vec{u}$ did not change.

Table 3 summarizes the speed ${\hat{U}}_{s}$ and relative error $\varepsilon_{r_{s}}$ that were computed for each input image, in which s∈{1.0,0.50,0.25} denotes the scaling factor, s=1.0 is the original scale, and *U* is the true speed value that was measured by the radar.

Recall that the resolution of the original images (s=1.0) was 6000×4000 pixels. However, it is important to notice that, except for the detection of the vanishing line, all visual information that was used by our approach was within the ROI in image space. The average resolution of the axis-aligned bounding box of the ROI at s=1.0 was only 151×1647 pixels. For the 0.5, 0.25, and 0.125 scaled images, the average resolutions of the ROI were 73×807, 36×406, and 17×202 pixels, respectively.

According to Table 3, most of the speeds that were calculated with a scale of 0.5 were close to the true speed, the relative errors were below 10% for 13 images, and there were eight cases between 10% and 50%. Image i16 was the least affected by the change in resolution, which presented a degradation of only 10% of the relative error compared to the original image. It was only not possible to estimate speeds for images i7 and i21. Among those that resulted in measurements, the worst case was image i12 with a 3500% degradation in the relative error. The mean and median degradations were 681.66% and 300%, respectively.

In total, it was not possible to estimate speeds using 43.5% of the images with a scale of 0.25. The relative errors for the remaining cases were below 13% for seven images and there were six cases between 18% and 70%. The mean relative error for images with a scale of 0.25 was 22.74%, while the mean and median degradations of the relative error concerning the original images were 1514.63% and 600%, respectively.

These results showed that the resolution of the ROI and, hence, the amount of information that was available to produce a quality edge image was critical to the performance of the proposed approach, since the edge image was used to provide the visual clues for the identification of the wave arms. In the practical use of this technique, automatic zooming could be used to increase the resolution of the ROI.

### 4.6. Resilience to Variations in JPG Compression Rate

In the last experiment, we analyzed the variation in estimated speeds as we changed the JPG image compression level. For each original image that was stored at 100% quality, we created copies at 90%, 75%, and 50% quality and applied the approach that was described in Section 2.

In Table 4 it is possible to observe that the estimated speed ${\hat{U}}_{100\%}$ did not change much compared to the lower resolution counterparts of the same image. Surprisingly, in images i1, i7, i14, i15, i16, i21, and i22, there was a decrease of 1 to 4% in the relative error of the estimates for images with 90%, 75%, or 50% quality. The biggest increase in relative error was 6% for image i18 at 75% quality. As mentioned before, image i18 (Figure 6g) was one of the noisy images that was captured under a scattered storm. In another 20 cases (images i3, i4, i5, i7, i13, i15, i17, i18, i20, and i22), the increase ranged from 1 to 5%. In 32 out of the 69 cases, the increase or decrease in the relative error was less than 1%.

The results that were presented in this section suggested that the proposed approach had a good resilience to the compression of the input image. We believe that the reason for this is the robustness of the edge detection technique that was used in our implementation.

## 5. Conclusions and Future Works

We presented a method for the estimation of vessel speed using single perspective projection images. The approach uses geometric constraints to remove perspective distortion from the images of traces that were left by a moving vessel and uses curve fitting and peak detection to identify troughs in the wave arms and natural constraints in components of Kelvin wakes in order to compute vessel speed.

We validated the measurements that were produced by our approach using the speeds that were obtained by a radar. The quality of the results was verified by the application of a statistical analysis on the estimated speeds. We also used error propagation along the computational chain to provide reliable confidence intervals that provided a notion of the quality of the speeds that were estimated from a single image and presented a study that could identify the set of input parameters that had more impact on the uncertainty of the estimated speeds. The statistical analysis revealed that the estimated speeds were accurate and precise. We believe that our algorithm could be used by autonomous vessels and for maritime surveillance using drones and smart lighthouses.

In order to consider the use of our technique in real situations, it is necessary to draw some recommendations:

1. Lighting conditions affect edge detection and the detection of wave arms. In our experiments, we had no problems in daylight, but it was not always possible to process images that were captured at dawn or dusk and our solution cannot be applied at night. The same applies to rain and fog;
2. Due to geographical restrictions in our experiments, we used images of the port and starboard of vessels that were traveling in the left and right directions in front of the camera and moving along a linear course at a (supposedly) constant speed. However, we believe that our approach is robust to variations in camera orientation since it was possible to see the troughs in the wake, even at the grazing angle;
3. As demonstrated in our experiments, well-defined capillary wakes due to wind and, possibly, those generated by nearby vessels may affect the Kelvin wake pattern. However, we believe that this is a problem that could be overcome by the detection of crossing wakes;
4. Since this method is to be applied to single images, the use of video could provide dozens of independent measurements per second, which could be combined to reduce error or eliminate spurious estimates;
5. Although we did not try this in our experiments, pre-processing the images to increase contrast could help in the detection of the wakes of slower vessels.

Unfortunately, the radar that we used could not automatically display information on the speed of small vessels, e.g., sailboats and fishing boats. This is because small vessels do not have to be equipped with an automatic identification system (AIS), which allows sensors to display the ship’s speed information. In addition, the social isolation that was imposed by the COVID-19 pandemic prevented us from having access to the radar to expand data acquisition. Even so, we were able to obtain speed information from several medium-sized vessels. The results showed that our approach was robust. It was validated by considering the measurements from standard nautical equipment and we believe that it could be applied to any vessel that leaves distinguishable wake patterns. As a direction for future work, we point to the investigation of the influence of climatic conditions to better analyze the few cases in which the results were not consistent. For this analysis, images must be systematically captured at different times of day, under various lighting conditions, in different weather conditions, at different wind speeds, and in all four seasons of the year. So, ideally, systematic captures need to take place over at least one year in order to obtain a wide range of image conditions.

Another direction for future work is to extend the analyses that were presented in this paper through the application of the ISO Guide to the Expression of Uncertainty of Measurement (GUM) [32].

The testbed implementation of our approach took approximately 12 s to process each image. Most of that time was used to compute the edge image using the RCF algorithm. Faster methods for calculating the edge images proved ineffective in enhancing the ship wakes. So, we aim to optimize this stage to calculate the vessel speed in real time. We are also exploring ways to work with super-resolution images in order to improve the quality of the information being included in the ROI and ways to use a sequence of images to calculate the vessel speed in each image and analyze the resulting speeds, although the central idea of this work was to use only one image. Our implementation will be made available after the publication of this paper.

## Figures and Tables

**Table 1:** Notation convention that is used in this article.

Notation	Meaning
I,B	Input and edge image, respectively
Π	Water plane
$x_{i}$	The *i*-th point in image space under homogeneous coordinates
$X_{i}$	The *i*-th point in world space under homogeneous coordinates
$x_{x_{i}},y_{x_{i}}$	Coordinates of point $x_{i}$ in image space
$X_{X_{i}},Y_{X_{i}},Z_{X_{i}}$	Coordinates of point $X_{i}$ in world space
l	Vector encoding the vanishing line in image space
$\vec{v}$	Vector in image space under homogeneous coordinates
$\vec{V}$	Vector in world space under homogeneous coordinates
M	An m×n matrix
$M^{-1}$	Inverse of a matrix M
$M^{T}$	Transpose of a matrix M

**Table 2:** Information regarding each captured image, where *U* denotes the speed that was measured by a radar (ground truth), $\hat{U}$ is the speed that was estimated by our approach using the troughs of the wave arms, and $\varepsilon_{a}$ and $\varepsilon_{r}$ are the absolute and relative errors of estimations, respectively. The subtable summarizes the results of Huillca and Fernandes [13].

Image	Vessel		Time (hh:mm)	Weather	Tide (Meters)	Speed (knots)		Error		Results of [13]	
	Model	Name				*U*	$\hat{U}$	$\varepsilon_{a}$	$\varepsilon_{r}$	$\hat{U}$	$\varepsilon_{r}$
i1	HSC	Fenix	10:09	Cloudy	0.30	18.2	18.339	0.139	0.008	17.234	0.053
i2	MC25	Apolo	10:12	Cloudy	0.30	20.6	21.533	0.933	0.045	25.467	0.236
i3	MC25	Apolo	10:13	Cloudy	0.30	20.5	20.609	0.109	0.005	26.629	0.299
i4	MC25	Neptuno	10:19	Cloudy	0.50	19.2	19.434	0.234	0.012	21.788	0.135
i5	MC25	Neptuno	10:21	Cloudy	0.50	17.3	16.683	0.617	0.036	20.346	0.176
i6	MC25	Neptuno	10:22	Cloudy	0.50	17.1	16.530	0.570	0.033	27.785	0.625
i7	Other	Escander Amazonas	10:28	Cloudy	0.60	09.2	19.262	10.062	1.094	17.326	0.883
i8	MC25	*Missing*	10:39	Cloudy	0.70	19.5	19.288	0.212	0.011	26.036	0.335
i9	HSC	Fenix	10:41	Cloudy	0.70	15.6	15.984	0.384	0.025	18.231	0.169
i10	HSC	Fenix	10:43	Cloudy	0.70	16.5	16.611	0.111	0.007	20.133	0.220
i11	MC25	*Missing*	11:08	Cloudy	0.50	20.4	20.771	0.371	0.018	26.436	0.296
i12	MC25	*Missing*	11:09	Cloudy	0.50	20.4	20.543	0.143	0.007	23.863	0.170
i13	MC25	*Missing*	11:12	Cloudy	0.50	17.3	16.846	0.454	0.026	23.259	0.344
i14	MC25	Zeus	11:42	Cloudy	0.70	19.6	20.533	0.933	0.048	26.982	0.377
i15	MC25	Neptuno	11:43	Cloudy	0.70	19.1	19.861	0.761	0.040	19.407	0.016
i16	MC25	Neptuno	12:10	Cloudy	0.90	20.2	22.120	1.920	0.095	25.951	0.285
i17	MC25	*Missing*	12:13	Cloudy	0.90	20.3	20.638	0.338	0.017	22.254	0.096
i18	MC25	Zeus	16:12	Scattered storm	1.10	18.9	19.617	0.717	0.038	22.086	0.169
i19	MC25	Zeus	16:50	Partly cloudy	0.70	17.5	17.342	0.158	0.009	22.249	0.271
i20	MC25	Zeus	16:51	Partly cloudy	0.70	17.6	17.808	0.208	0.012	19.947	0.133
i21	MC25	Zeus	16:51	Partly cloudy	0.70	17.8	18.947	1.147	0.064	25.107	0.410
i22	MC25	*Missing*	17:01	Partly cloudy	0.70	20.3	16.490	3.810	0.188	23.448	0.155
i23	MC25	Zeus	17:21	Partly cloudy	0.50	16.8	16.084	0.716	0.043	21.008	0.251

**Table 3:** Variation in the estimated speeds as a function of image resolution: *U* is the speed that was measured by the radar; ${\hat{U}}_{s}$ and $\varepsilon_{r_{s}}$ are the speed that was estimated by our approach and the relative error that was obtained using an input image at a scale of *s*, respectively; and s=1.0 for the original images.

Image	Speed (knots)				Relative Error		
	U	${\hat{U}}_{1.00}$	${\hat{U}}_{0.50}$	${\hat{U}}_{0.25}$	$\varepsilon_{r_{1.00}}$	$\varepsilon_{r_{0.50}}$	$\varepsilon_{r_{0.25}}$
i1	18.2	18.34	18.75	–	0.01	0.03	–
i2	20.6	21.53	22.39	21.82	0.05	0.09	0.06
i3	20.5	20.61	23.53	13.64	0.01	0.15	0.33
i4	19.2	19.43	20.71	19.36	0.01	0.08	0.01
i5	17.3	16.68	19.10	13.96	0.04	0.10	0.19
i6	17.1	16.53	17.93	17.86	0.03	0.05	0.04
i7	09.2	19.26	–	–	1.09	–	–
i8	19.5	19.29	19.30	21.75	0.01	0.01	0.12
i9	15.6	15.98	19.38	–	0.02	0.24	–
i10	16.5	16.61	16.15	15.38	0.01	0.02	0.07
i11	20.4	20.77	22.24	6.40	0.02	0.09	0.69
i12	20.4	20.54	13.32	10.45	0.01	0.35	0.49
i13	17.3	16.85	18.78	–	0.03	0.09	–
i14	19.6	20.53	19.50	–	0.05	0.01	–
i15	19.1	19.86	11.08	–	0.04	0.42	–
i16	20.2	22.12	20.09	–	0.10	0.01	–
i17	20.3	20.64	18.95	–	0.02	0.07	–
i18	18.9	19.62	22.07	14.48	0.04	0.17	0.23
i19	17.5	17.34	21.46	18.58	0.01	0.23	0.06
i20	17.6	17.81	19.24	10.54	0.01	0.09	0.40
i21	17.8	18.95	–	–	0.06	–	–
i22	20.3	16.49	10.22	14.99	0.19	0.50	0.26
i23	16.8	16.08	17.09	–	0.04	0.02	–

**Table 4:** Variation in estimated speeds as a function of image compression: *U* is the speed that was measured by the radar; ${\hat{U}}_{q}$ and $\varepsilon_{r_{q}}$ are the speed that was estimated by our approach and the relative error of using an image at quality compression *q*, respectively; and q=100% for the original images.

Image	Speed (knots)					Relative Error			
	U	${\hat{U}}_{100\%}$	${\hat{U}}_{90\%}$	${\hat{U}}_{75\%}$	${\hat{U}}_{50\%}$	$\varepsilon_{q_{100\%}}$	$\varepsilon_{q_{90\%}}$	$\varepsilon_{q_{75\%}}$	$\varepsilon_{q_{50\%}}$
i1	18.2	18.34	18.21	18.21	18.21	0.0077	0.0005	0.0005	0.0005
i2	20.6	21.52	21.48	21.48	21.48	0.0447	0.0427	0.0427	0.0427
i3	20.5	20.55	20.52	20.42	20.00	0.0024	0.0010	0.0039	0.0244
i4	19.2	19.63	19.93	19.93	20.27	0.0224	0.0380	0.0380	0.0557
i5	17.3	16.68	16.34	16.34	16.34	0.0358	0.0555	0.0555	0.0555
i6	17.1	16.53	16.54	16.54	16.54	0.0333	0.0327	0.0327	0.0327
i7	9.2	19.26	19.32	19.32	19.12	1.0935	1.1000	1.1000	1.0783
i8	19.5	19.29	19.28	19.28	19.28	0.0108	0.0113	0.0113	0.0113
i9	15.6	16.07	16.09	16.09	16.09	0.0301	0.0314	0.0314	0.0314
i10	16.5	16.61	16.55	16.55	16.55	0.0067	0.0030	0.0030	0.0030
i11	20.4	20.77	20.83	20.83	20.83	0.0181	0.0211	0.0211	0.0211
i12	20.4	21.04	21.10	21.10	21.03	0.0314	0.0343	0.0343	0.0309
i13	17.3	16.85	16.82	16.00	15.99	0.0260	0.0277	0.0751	0.0757
i14	19.6	20.38	20.17	20.21	20.21	0.0398	0.0291	0.0311	0.0311
i15	19.1	19.86	19.70	19.73	19.70	0.0398	0.0314	0.0330	0.0314
i16	20.2	21.42	21.22	21.76	21.22	0.0604	0.0505	0.0772	0.0505
i17	20.3	20.64	20.62	20.54	20.82	0.0167	0.0158	0.0118	0.0256
i18	18.9	19.62	20.29	20.80	20.61	0.0381	0.0735	0.1005	0.0905
i19	17.5	17.34	17.40	17.40	17.40	0.0091	0.0057	0.0057	0.0057
i20	17.6	17.72	16.95	17.87	17.12	0.0068	0.0369	0.0153	0.0273
i21	17.8	18.99	18.36	18.36	18.36	0.0669	0.0315	0.0315	0.0315
i22	20.3	17.02	17.25	17.25	6.80	0.1616	0.1502	0.1502	0.6650
i23	16.8	16.96	16.99	16.99	16.99	0.0095	0.0113	0.0113	0.0113

## Data Availability

Not applicable.

## Appendix A. Partial Derivatives for Error Propagation

Section 3 shows that we used first-order error propagation to compute the standard uncertainty of the estimated vessel speed ($\sigma_{U}$) using the uncertainties of the experimental input variables in the dataset ϑ as the sandwich product of the Jacobian matrix $\nabla_{U}$ and the covariance matrix $\Lambda_{\vartheta }$ (9). This appendix includes the expressions that were used to compute the partial derivatives in $\nabla_{U}$ using the chain rule. Here, we present the partial derivatives for the variables of the computational chain that is illustrated in Figure 5.

*Vanishing line l*. According to (1), the vanishing line of the water surface was computed from points $p_{l}$ and $p_{r}$. Thus:

$$\frac{\partial l}{\partial \vartheta }=\left(\begin{array}{c} \frac{\partial A}{\partial \vartheta }\\ \frac{\partial B}{\partial \vartheta }\\ \frac{\partial C}{\partial \vartheta } \end{array}\right)=\left(\begin{array}{c} \frac{\partial y_{p_{l}}}{\partial \vartheta }-\frac{\partial y_{p_{r}}}{\partial \vartheta }\\ 0\\ \left(1-W\right)\frac{\partial y_{p_{l}}}{\partial \vartheta } \end{array}\right),$$

where $\frac{\partial y_{p_{l}}}{\partial \vartheta }$ and $\frac{\partial y_{p_{r}}}{\partial \vartheta }$ are 1 when the derivatives are taken with respect to $y_{p_{l}}$ and $\partial y_{p_{r}}$, respectively, and are otherwise 0.

*Camera height h*. In this work, the camera height was given by (2). The partial derivative of *h* was:

$$\frac{\partial h}{\partial \vartheta }=\frac{\partial h_{g}}{\partial \vartheta }+\delta \frac{\partial h_{f}}{\partial \vartheta }+\frac{\partial h_{t}}{\partial \vartheta }-\frac{\partial h_{s}}{\partial \vartheta }.$$

where $h_{g}$, $h_{f}$, $h_{t}$, and $h_{s}$ are used as input; thus, their partial derivatives are 1 when taken with respect to themselves and are otherwise 0.

*Rotation matrix R*. It was easier to obtain the derivatives of R by considering its columns ${\vec{R}}_{1}$, ${\vec{R}}_{2}$, and ${\vec{R}}_{3}$ separately. In this case:

$$\frac{\partial R}{\partial \vartheta }=\left(\frac{\partial {\vec{R}}_{1}}{\partial \vartheta },\frac{\partial {\vec{R}}_{2}}{\partial \vartheta },\frac{\partial {\vec{R}}_{3}}{\partial \vartheta }\right),$$

where

$$\begin{array}{cc} \frac{\partial {\vec{R}}_{1}}{\partial \vartheta } & ={\vec{R}}_{3}\times \frac{\partial {\vec{R}}_{2}}{\partial \vartheta }+\frac{\partial {\vec{R}}_{3}}{\partial \vartheta }\times {\vec{R}}_{2},\\ \frac{\partial {\vec{R}}_{2}}{\partial \vartheta } & =\frac{Z_{{\vec{R}}_{3}}\frac{\partial Y_{{\vec{R}}_{3}}}{\partial \vartheta }-\frac{\partial Z_{{\vec{R}}_{3}}}{\partial \vartheta }Y_{{\vec{R}}_{3}}}{{\left(Y_{{\vec{R}}_{3}}^{2}+Z_{{\vec{R}}_{3}}^{2}\right)}^{\frac{3}{2}}}{\left(0,Y_{{\vec{R}}_{3}},Z_{{\vec{R}}_{3}}\right)}^{T},\;\mathrm{and}\\ \frac{\partial {\vec{R}}_{3}}{\partial \vartheta } & =\pm \frac{1}{{\left(X_{\vec{L}}^{2}+Y_{\vec{L}}^{2}+Z_{\vec{L}}^{2}\right)}^{\frac{3}{2}}}\left(\begin{array}{c} \left(Y_{\vec{L}}^{2}+Z_{\vec{L}}^{2}\right)\frac{\partial X_{\vec{L}}}{\partial \vartheta }-X_{\vec{L}}\left(Y_{\vec{L}}\frac{\partial Y_{\vec{L}}}{\partial \vartheta }+Z_{\vec{L}}\frac{\partial Z_{\vec{L}}}{\partial \vartheta }\right)\\ \left(X_{\vec{L}}^{2}+Z_{\vec{L}}^{2}\right)\frac{\partial Y_{\vec{L}}}{\partial \vartheta }-Y_{\vec{L}}\left(X_{\vec{L}}\frac{\partial X_{\vec{L}}}{\partial \vartheta }+Z_{\vec{L}}\frac{\partial Z_{\vec{L}}}{\partial \vartheta }\right)\\ \left(X_{\vec{L}}^{2}+Y_{\vec{L}}^{2}\right)\frac{\partial Z_{\vec{L}}}{\partial \vartheta }-Z_{\vec{L}}\left(X_{\vec{L}}\frac{\partial X_{\vec{L}}}{\partial \vartheta }+Y_{\vec{L}}\frac{\partial Y_{\vec{L}}}{\partial \vartheta }\right) \end{array}\right). \end{array}$$

The orientation of $\frac{\partial {\vec{R}}_{3}}{\partial \vartheta }$ was set according to the up function. Here, $\vec{L}={\left(X_{\vec{L}},Y_{\vec{L}},Z_{\vec{L}}\right)}^{T}=K^{T}l$ was an auxiliary variable with:

$$\frac{\partial \vec{L}}{\partial \vartheta }=\left(\begin{array}{c} \frac{\partial X_{\vec{L}}}{\partial \vartheta }\\ \frac{\partial Y_{\vec{L}}}{\partial \vartheta }\\ \frac{\partial Z_{\vec{L}}}{\partial \vartheta } \end{array}\right)=\left(\begin{array}{c} \alpha_{x}\frac{\partial A}{\partial \vartheta }\\ \gamma \frac{\partial A}{\partial \vartheta }\\ \frac{\partial C}{\partial \vartheta }+x_{o}\frac{\partial A}{\partial \vartheta } \end{array}\right).$$

*Matrices M and $M^{-1}$*. We assumed that the intrinsic parameters of the camera had no uncertainty. So, the derivatives of M were given by the multiplication of a constant matrix K and $\frac{\partial R}{\partial \vartheta }$:

$$\frac{\partial M}{\partial \vartheta }=\left(\begin{array}{ccc} \frac{\partial m_{11}}{\partial \vartheta } & \frac{\partial m_{12}}{\partial \vartheta } & \frac{\partial m_{13}}{\partial \vartheta }\\ \frac{\partial m_{21}}{\partial \vartheta } & \frac{\partial m_{22}}{\partial \vartheta } & \frac{\partial m_{23}}{\partial \vartheta }\\ \frac{\partial m_{31}}{\partial \vartheta } & \frac{\partial m_{32}}{\partial \vartheta } & \frac{\partial m_{33}}{\partial \vartheta } \end{array}\right)=K\frac{\partial R}{\partial \vartheta }.$$

The derivatives of the inverse of M were:

$$\frac{\partial M^{-1}}{\partial \vartheta }=-M^{-1}\frac{\partial M}{\partial \vartheta }M^{-1}.$$

*Direction $\vec{D}$*. The ray from the camera center to the reference point $X_{1}$ required the input of point $x_{1}$ and the computation of $M^{-1}$ (see (3)), from which it followed:

$$\frac{\partial \vec{D}}{\partial \vartheta }=\left(\begin{array}{c} \frac{\partial X_{\vec{D}}}{\partial \vartheta }\\ \frac{\partial Y_{\vec{D}}}{\partial \vartheta }\\ \frac{\partial Z_{\vec{D}}}{\partial \vartheta } \end{array}\right)=M^{-1}\frac{\partial x_{1}}{\partial \vartheta }+\frac{\partial M^{-1}}{\partial \vartheta }x_{1},\;\mathrm{were}\;\frac{\partial x_{1}}{\partial \vartheta }={\left(\frac{\partial x_{x_{1}}}{\partial \vartheta },\frac{\partial y_{x_{1}}}{\partial \vartheta },0\right)}^{T}$$

where $\frac{\partial x_{x_{1}}}{\partial \vartheta }$ and $\frac{\partial y_{x_{1}}}{\partial \vartheta }$ are 1 when taken with respect to themselves and are otherwise 0.

*ROI corner $X_{1}$ in world space*. After computing the direction $\vec{D}$, the computation of the derivatives of $X_{1}$ was straightforward:

$$\frac{\partial X_{1}}{\partial \vartheta }=\left(\begin{array}{c} \frac{\partial X_{X_{1}}}{\partial \vartheta }\\ \frac{\partial Y_{X_{1}}}{\partial \vartheta }\\ \frac{\partial Z_{X_{1}}}{\partial \vartheta }\\ \frac{\partial W_{X_{1}}}{\partial \vartheta } \end{array}\right)=\frac{1}{Z_{\vec{D}}^{2}}\left(\begin{array}{c} hX_{\vec{D}}\frac{\partial Z_{\vec{D}}}{\partial \vartheta }-Z_{\vec{D}}\left(X_{\vec{D}}\frac{\partial h}{\partial \vartheta }+h\frac{\partial X_{\vec{D}}}{\partial \vartheta }\right)\\ hY_{\vec{D}}\frac{\partial Z_{\vec{D}}}{\partial \vartheta }-Z_{\vec{D}}\left(Y_{\vec{D}}\frac{\partial h}{\partial \vartheta }+h\frac{\partial Y_{\vec{D}}}{\partial \vartheta }\right)\\ 0\\ 0 \end{array}\right).$$

*Direction $\vec{E}$*. The back projection of the improper point that was encoding the direction of the vessel in I was $\vec{E}=M^{-1}\vec{u}$, which led to:

$$\frac{\partial \vec{E}}{\partial \vartheta }=\left(\begin{array}{c} \frac{\partial X_{\vec{E}}}{\partial \vartheta }\\ \frac{\partial Y_{\vec{E}}}{\partial \vartheta }\\ \frac{\partial Z_{\vec{E}}}{\partial \vartheta } \end{array}\right)=M^{-1}\frac{\partial \vec{u}}{\partial \vartheta }+\frac{\partial M^{-1}}{\partial \vartheta }\vec{u},\;\mathrm{where}\;\frac{\partial \vec{u}}{\partial \vartheta }=\left(\begin{array}{c} \frac{\partial x_{\vec{u}}}{\partial \vartheta }\\ \frac{\partial v_{\vec{u}}}{\partial \vartheta }\\ \frac{\partial w_{\vec{u}}}{\partial \vartheta } \end{array}\right)=\frac{\partial \phi_{\vec{u}}}{\partial \vartheta }\left(\begin{array}{c} -\sin\phi_{\vec{u}}\\ \cos\phi_{\vec{u}}\\ 0 \end{array}\right).$$

where, as an input variable, the derivative of $\phi_{\vec{u}}$ is 1 with respect to itself and 0 for any other case.

*Vessel direction $\vec{U}$ in world space*. We let A be a constant matrix that encoded the orthogonal projection onto Π. We computed $\vec{U}$ by applying A to $\vec{E}$ and normalizing the result to unit length:

$$\vec{U}={\left(X_{\vec{U}},Y_{\vec{U}},Z_{\vec{U}},W_{\vec{U}}\right)}^{T}=\mathrm{unit}\left(A\vec{E}\right)=\frac{1}{\sqrt{X_{\vec{E}}^{2}+Y_{\vec{E}}^{2}}}{\left(X_{\vec{E}},Y_{\vec{E}},0,0\right)}^{T}.$$

The partial derivatives of $\vec{U}$ with respect to ϑ were:

$$\frac{\partial \vec{U}}{\partial \vartheta }={\left(\frac{\partial X_{\vec{U}}}{\partial \vartheta },\frac{\partial Y_{\vec{U}}}{\partial \vartheta },\frac{\partial Z_{\vec{U}}}{\partial \vartheta },\frac{\partial W_{\vec{U}}}{\partial \vartheta }\right)}^{T}=\frac{X_{\vec{E}}\frac{\partial X_{\vec{E}}}{\partial \vartheta }+Y_{\vec{E}}\frac{\partial Y_{\vec{E}}}{\partial \vartheta }}{{\left(X_{\vec{E}}^{2}+Y_{\vec{E}}^{2}\right)}^{\frac{3}{2}}}{\left(-X_{\vec{E}},-Y_{\vec{E}},0,0\right)}^{T}.$$

*Direction $\vec{V}$*. Vector $\vec{V}$ was computed by rotating $\vec{U}$ on the mean water plane by π/2 radians:

$$\vec{V}={\left(X_{\vec{V}},Y_{\vec{V}},Z_{\vec{V}},W_{\vec{V}}\right)}^{T}=B\vec{U}={\left(-Y_{\vec{U}},X_{\vec{U}},0,0\right)}^{T},$$

where B is a constant rotation matrix. The derivatives of $\vec{V}$ came from $\vec{U}$:

$$\frac{\partial \vec{V}}{\partial \vartheta }={\left(\frac{\partial X_{\vec{V}}}{\partial \vartheta },\frac{\partial Y_{\vec{V}}}{\partial \vartheta },\frac{\partial Z_{\vec{V}}}{\partial \vartheta },\frac{\partial W_{\vec{V}}}{\partial \vartheta }\right)}^{T}={\left(-\frac{\partial Y_{\vec{U}}}{\partial \vartheta },\frac{\partial X_{\vec{U}}}{\partial \vartheta },0,0\right)}^{T}.$$

*ROI corners ${\vec{X}}_{2}$ and ${\vec{X}}_{3}$ in world space*. Equation (Section 2.2) showed how to compute corners $X_{k}$, for k={2,3,4}, from the intermediate variables $X_{1}$, $\vec{U}$, and $\vec{V}$. However, only $X_{2}$ and $X_{3}$ were needed in the remainder of the computational flow. Their derivatives were:

$$\begin{array}{cc} \frac{\partial X_{2}}{\partial \vartheta } & ={\left(\frac{\partial X_{X_{2}}}{\partial \vartheta },\frac{\partial Y_{X_{2}}}{\partial \vartheta },\frac{\partial Z_{X_{2}}}{\partial \vartheta },\frac{\partial W_{X_{2}}}{\partial \vartheta }\right)}^{T}=\frac{\partial X_{1}}{\partial \vartheta }-W_{ROI}\frac{\partial \vec{U}}{\partial \vartheta },\;\mathrm{and}\\ \frac{\partial X_{3}}{\partial \vartheta } & ={\left(\frac{\partial X_{X_{3}}}{\partial \vartheta },\frac{\partial Y_{X_{3}}}{\partial \vartheta },\frac{\partial Z_{X_{3}}}{\partial \vartheta },\frac{\partial W_{X_{3}}}{\partial \vartheta }\right)}^{T}=\frac{\partial X_{1}}{\partial \vartheta }-H_{ROI}\frac{\partial \vec{V}}{\partial \vartheta }. \end{array}$$

*Camera matrix P*. By rewriting the camera matrix as:

$$P=\left(\begin{array}{c} M|-M\tilde{C} \end{array}\right)=\left(\begin{array}{cccc} m_{11} & m_{12} & m_{13} & -h\;m_{13}\\ m_{21} & m_{22} & m_{23} & -h\;m_{23}\\ m_{31} & m_{32} & m_{33} & -h\;m_{33} \end{array}\right),$$

it was easy to see that the partial derivatives of its components were:

$$\frac{\partial P}{\partial \vartheta }=\left(\begin{array}{cccc} \frac{\partial p_{11}}{\partial \vartheta } & \frac{\partial p_{12}}{\partial \vartheta } & \frac{\partial p_{13}}{\partial \vartheta } & \frac{\partial p_{14}}{\partial \vartheta }\\ \frac{\partial p_{21}}{\partial \vartheta } & \frac{\partial p_{22}}{\partial \vartheta } & \frac{\partial p_{23}}{\partial \vartheta } & \frac{\partial p_{24}}{\partial \vartheta }\\ \frac{\partial p_{31}}{\partial \vartheta } & \frac{\partial p_{32}}{\partial \vartheta } & \frac{\partial p_{33}}{\partial \vartheta } & \frac{\partial p_{34}}{\partial \vartheta } \end{array}\right)=\left(\begin{array}{cc} \frac{\partial M}{\partial \vartheta } & \begin{array}{c} -\left(h\frac{\partial m_{13}}{\partial \vartheta }+m_{13}\frac{\partial h}{\partial \vartheta }\right)\\ -\left(h\frac{\partial m_{23}}{\partial \vartheta }+m_{23}\frac{\partial h}{\partial \vartheta }\right)\\ -\left(h\frac{\partial m_{33}}{\partial \vartheta }+m_{33}\frac{\partial h}{\partial \vartheta }\right) \end{array} \end{array}\right).$$

*ROI corners $x_{2}$ and $x_{3}$ in image space*. Recall from (5) that, except for the reference corner $x_{1}$, the corners $x_{k}$ were computed by projecting $X_{k}$ from world space onto image space. The partial derivatives of those corners were:

$$\frac{\partial x_{k}}{\partial \vartheta }=\left(\begin{array}{c} \frac{\partial x_{x_{k}}}{\partial \vartheta }\\ \frac{\partial y_{x_{k}}}{\partial \vartheta }\\ \frac{\partial w_{x_{k}}}{\partial \vartheta } \end{array}\right)=\left(\begin{array}{c} X_{X_{k}}\frac{\partial m_{11}}{\partial \vartheta }+m_{11}\frac{\partial X_{X_{k}}}{\partial \vartheta }+Y_{X_{k}}\frac{\partial m_{12}}{\partial \vartheta }+m_{12}\frac{\partial Y_{X_{k}}}{\partial \vartheta }+\frac{\partial p_{14}}{\partial \vartheta }\\ X_{X_{k}}\frac{\partial m_{21}}{\partial \vartheta }+m_{21}\frac{\partial X_{X_{k}}}{\partial \vartheta }+Y_{X_{k}}\frac{\partial m_{22}}{\partial \vartheta }+m_{22}\frac{\partial Y_{X_{k}}}{\partial \vartheta }+\frac{\partial p_{24}}{\partial \vartheta }\\ X_{X_{k}}\frac{\partial m_{31}}{\partial \vartheta }+m_{31}\frac{\partial X_{X_{k}}}{\partial \vartheta }+Y_{X_{k}}\frac{\partial m_{32}}{\partial \vartheta }+m_{32}\frac{\partial Y_{X_{k}}}{\partial \vartheta }+\frac{\partial p_{34}}{\partial \vartheta } \end{array}\right).$$

*Homographic matrix H*. In (6), H was computed by multiplying the matrices $H_{A}$ and $H_{l}$, where the former was defined by the coefficients of the right-hand null space vector $\vec{v}$ and the later was related to the vanishing line l. In its final form:

$$H=\left(\begin{array}{ccc} h_{11} & h_{12} & h_{13}\\ h_{21} & h_{22} & h_{23}\\ h_{31} & h_{33} & h_{33} \end{array}\right)=H_{A}H_{l}.$$

The partial derivatives of H with respect to ϑ were:

$$\frac{\partial H}{\partial \vartheta }=\left(\begin{array}{ccc} \frac{\partial h_{11}}{\partial \vartheta } & \frac{\partial h_{12}}{\partial \vartheta } & \frac{\partial h_{13}}{\partial \vartheta }\\ \frac{\partial h_{21}}{\partial \vartheta } & \frac{\partial h_{22}}{\partial \vartheta } & \frac{\partial h_{23}}{\partial \vartheta }\\ \frac{\partial h_{31}}{\partial \vartheta } & \frac{\partial h_{32}}{\partial \vartheta } & \frac{\partial h_{33}}{\partial \vartheta } \end{array}\right)=H_{A}\frac{\partial H_{l}}{\partial \vartheta }+\frac{\partial H_{A}}{\partial \vartheta }H_{l},$$

where

$$\begin{array}{cc} \frac{\partial H_{A}}{\partial \vartheta } & =\left(\begin{array}{ccc} \frac{v_{33}\frac{\partial v_{11}}{\partial \vartheta }-v_{11}\frac{\partial v_{33}}{\partial \vartheta }}{v_{33}^{2}} & \frac{v_{33}\frac{\partial v_{12}}{\partial \vartheta }-v_{12}\frac{\partial v_{33}}{\partial \vartheta }}{v_{33}^{2}} & \frac{v_{33}\frac{\partial v_{13}}{\partial \vartheta }-v_{13}\frac{\partial v_{33}}{\partial \vartheta }}{v_{33}^{2}}\\ \frac{v_{33}\frac{\partial v_{21}}{\partial \vartheta }-v_{21}\frac{\partial v_{33}}{\partial \vartheta }}{v_{33}^{2}} & \frac{v_{33}\frac{\partial v_{22}}{\partial \vartheta }-v_{22}\frac{\partial v_{33}}{\partial \vartheta }}{v_{33}^{2}} & \frac{v_{33}\frac{\partial v_{23}}{\partial \vartheta }-v_{23}\frac{\partial v_{33}}{\partial \vartheta }}{v_{33}^{2}}\\ 0 & 0 & 1 \end{array}\right),\;\mathrm{and}\\ \frac{\partial H_{l}}{\partial \vartheta } & =\left(\begin{array}{ccc} 1 & 0 & 0\\ 0 & 1 & 0\\ \frac{\partial A}{\partial \vartheta } & 0 & \frac{\partial C}{\partial \vartheta } \end{array}\right). \end{array}$$

The procedure to obtain the derivatives of $\vec{v}$ from the singular value decomposition can be found in the literature [33].

*Location $c_{1}$ and $c_{2}$ of troughs in the rectified ROI*. We were not able to estimate the uncertainty of the location of the maximum and minimum of the smooth curve that was computed by the LOWESS algorithm. Our approach to not interrupting the uncertainty propagation of the computational chain while it was estimating $c_{1}$ and $c_{2}$ in the rectified ROI was:

1. Take the coordinates of the troughs in the rectified ROI;
2. Map them onto I using the inverse homography $H^{-1}$, thereby obtaining $c_{1}^{\prime }$ and $c_{2}^{\prime }$:
  $$c_{j}^{\prime }={\left(x_{c_{j}^{\prime }},y_{c_{j}^{\prime }},w_{c_{j}^{\prime }}\right)}^{T}=H^{-1}{\left(x_{c_{j}},y_{c_{j}},1\right)}^{T};$$
3. Consider that $c_{1}^{\prime }$ and $c_{2}^{\prime }$ are variables without uncertainty (see the pentagons in Figure 5);
4. Transport the uncertainty that are propagated on H onto $c_{1}$ and $c_{2}$ by mapping $c_{1}^{\prime }$ and $c_{2}^{\prime }$ back to the rectified ROI using:
  $$c_{j}={\left(x_{c_{j}},y_{c_{j}},1\right)}^{T}=Hc_{j}^{\prime }=H{\left(x_{c_{j}^{\prime }},y_{c_{j}^{\prime }},w_{c_{j}^{\prime }}\right)}^{T}.$$

The derivatives of $c_{j}$, for j∈{1,2}, were:

$$\frac{\partial c_{j}}{\partial \vartheta }=\left(\begin{array}{c} \frac{\partial x_{c_{j}}}{\partial \vartheta }\\ \frac{\partial y_{c_{j}}}{\partial \vartheta }\\ \frac{\partial w_{c_{j}}}{\partial \vartheta } \end{array}\right)=\left(\begin{array}{c} \frac{\partial h_{11}}{\partial \vartheta }x_{c_{j}^{\prime }}+\frac{\partial h_{12}}{\partial \vartheta }y_{c_{j}^{\prime }}+\frac{\partial h_{13}}{\partial \vartheta }w_{c_{j}^{\prime }}\\ \frac{\partial h_{21}}{\partial \vartheta }x_{c_{j}^{\prime }}+\frac{\partial h_{22}}{\partial \vartheta }y_{c_{j}^{\prime }}+\frac{\partial h_{23}}{\partial \vartheta }w_{c_{j}^{\prime }}\\ \frac{\partial h_{31}}{\partial \vartheta }x_{c_{j}^{\prime }}+\frac{\partial h_{32}}{\partial \vartheta }y_{c_{j}^{\prime }}+\frac{\partial h_{33}}{\partial \vartheta }w_{c_{j}^{\prime }} \end{array}\right).$$

*Vessel speed U*. By replacing *D* and λ (7) in (8), *U* was written in terms of a constant term *r* that multiplied the square root of the Euclidean distance between points $c_{1}$ and $c_{2}$:

$$U=r\sqrt[{4}]{{\left(x_{c_{2}}-x_{c_{1}}\right)}^{2}+{\left(y_{c_{2}}-y_{c_{1}}\right)}^{2}},\;\mathrm{where}\;r=1.944\sqrt{\frac{g\sqrt{3}}{4\pi }}.$$

The components of $\nabla_{U}$ (10) were computed as:

$$\frac{\partial U}{\partial \vartheta }=\frac{r\left(\left(x_{c_{1}}-x_{c_{2}}\right)\left(\frac{\partial x_{c_{1}}}{\partial \vartheta }-\frac{\partial x_{c_{2}}}{\partial \vartheta }\right)+\left(y_{c_{1}}-y_{c_{2}}\right)\left(\frac{\partial y_{c_{1}}}{\partial \vartheta }-\frac{\partial y_{c_{2}}}{\partial \vartheta }\right)\right)}{2{\left({\left(x_{c_{1}}-x_{c_{2}}\right)}^{2}+{\left(y_{c_{1}}-y_{c_{2}}\right)}^{2}\right)}^{\frac{3}{4}}}.$$
